# Availability of Injectable Antimicrobial Drugs for Gonorrhea and Syphilis, United States, 2016

**DOI:** 10.3201/eid2511.190764

**Published:** 2019-11

**Authors:** William S. Pearson, Donald K. Cherry, Jami S. Leichliter, Laura H. Bachmann, Nicole A. Cummings, Matthew Hogben

**Affiliations:** Centers for Disease Control and Prevention, Atlanta, Georgia, USA (W.S. Pearson, J.S. Leichliter, L.H. Bachmann, M. Hogben);; Centers for Disease Control and Prevention, Hyattsville, Maryland, USA (D.K. Cherry, N.A. Cummings)

**Keywords:** antibiotic, gonorrhea, syphilis, *Neisseria gonorrheoae*, *Treponema pallidum*, access to care, drug availability, antimicrobial drugs, United States, bacteria, STIs, sexually transmitted infections, injectable antimicrobial drugs, ceftriaxone, penicillin G benzathine, patient-centered medical homes

## Abstract

We estimated the availability of the injectable antimicrobial drugs recommended for point-of-care treatment of gonorrhea and syphilis among US physicians who evaluated patients with sexually transmitted infections in 2016. Most physicians did not have these drugs available on-site. Further research is needed to determine the reasons for the unavailability of these drugs.

Rates of sexually transmitted infections (STIs) are on the rise in the United States. Compared with the incidence in 2013, the numbers of reported gonorrhea (+75%) and syphilis (primary and secondary, +153%) cases in 2017 were dramatically increased (*1*). Timely (optimally same-day) treatment of bacterial STIs with a highly effective regimen is critical for national STI control efforts and can help mitigate the development of drug resistance, a particularly pertinent issue for *Neisseria gonorrhoeae* (*2*). In addition, the high number of congenital syphilis cases in the United States in 2017 highlights the need for efficient and effective treatment of *Treponema pallidum* infection (*3*).

The recommended first-line treatment for uncomplicated gonorrhea is intramuscular ceftriaxone (250 mg) and for primary and secondary syphilis is intramuscular penicillin G benzathine (2.5 million units) (*4*). On-site access to these injectable medications in clinics facilitates point-of-care treatment and helps mitigate drug resistance to *N. gonorrhoeae* and *T. pallidum* when these drugs are used instead of the oral antimicrobial drug alternatives. Therefore, we set out to determine a nationally representative estimate of the availability of these medications and examine if differences existed by geographic location and between offices designated and not designated as patient-centered medical homes (PCMHs).

We used the 2016 Physician Induction File of the National Ambulatory Medical Care Survey (*5*) to assess the number of physicians who treat patients with STIs and had injectable antimicrobial drugs available on-site. The Physician Induction File is a nationally representative survey of office-based, nonfederal physicians in the United States and includes information regarding practice characteristics and habits. Use of these data is restricted, and access is facilitated through the Research Data Center, National Center for Health Statistics, Centers for Disease Control and Prevention, in Hyattsville, Maryland, USA. A total of 1,030 physicians (46.2% unweighted response rate), representing an estimated of 330,581 (95% CI 326,994–334,168) physicians in the United States, completed the Physician Induction File in 2016. In this survey, physicians who reported evaluating or treating patients for STIs were asked which antimicrobial drugs they had available on-site for same-day management of gonorrhea and syphilis, including intramuscular ceftriaxone and penicillin G benzathine at the recommended doses. We determined national estimates of reported on-site, same-day availability for these antimicrobial drugs and stratified results by PCMH designation and US region. We used multiple logistic regression models to determine if PCMH designation and region were predictive of on-site availability of these 2 medications.

An estimated 45.2% (149,483, 95% CI 138,850–160,116) of office-based physicians indicated that they evaluate patients for STIs in their offices. Of these, 77.9% (116,479, 95% CI 105,360–127,598) reported not having penicillin G benzathine available on-site and 56.1% (83,827, 95% CI 73,709–93,945) reported not having ceftriaxone. Access to both of these drugs was generally higher in the South (Table). Physicians in offices not designated PCMHs were more likely than those in offices designated PCMHs to report lacking on-site availability of ceftriaxone (odds ratio 2.03, 95% CI 1.15–3.57) and penicillin G benzathine (odds ratio 3.20, 95% CI 1.63–6.29) (Table).

**Table:** On-site availability of drugs to manage gonorrhea and syphilis and likelihood of drug nonavailability by type and location of clinic, United States, 2016*

Category	Weighted population, no. (95% CI)	On-site drug availability, % (95% CI)			Likelihood of nonavailability†	
		Yes	No		β	OR (95% CI)
Ceftriaxone, 250 mg, intramuscular						
PCMH designation‡						
Yes	44,028 (35,296–52,760)	54.4 (42.1–66.3)	45.6 (33.7–57.9)		Referent	Referent
No	89,381 (79,356–99,406)	37.3 (30.0–45.0)	62.7 (55.0–70.0)		0.71	2.03 (1.15–3.57)
Region of office§						
Northeast	33,384 (28,666–38,102)	34.3 (22.7–47.5)	65.7 (52.5–77.3)		Referent	Referent
South	45,574 (39,694–51,454)	49.0 (38.4–59.6)	51.0 (40.4–61.6)		−0.63	0.53 (0.27–1.06)
Midwest	33,296 (28,374–38,218)	41.4 (28.9–54.8)	58.6 (45.2–71.1)		−0.28	0.76 (0.35–1.64)
West	37,228 (31,038–43,418)	48.6 (35.8–61.6)	51.4 (38.4–64.2)		−0.62	0.54 (0.26–1.14)
Penicillin G benzathine, 2.5 million units, intramuscular						
PCMH designation‡						
Yes	44,028 (35,296–52,760	34.2 (23.2–46.6)	65.8 (53.4–76.8)		Referent	Referent
No	89,381 (79,356–99,406)	14.6 (9.5–21.1)	85.4 (78.9–90.5)		1.16	3.20 (1.63–6.29)
Region of office§						
Northeast	33,384 (28,666–38,102)	17.8 (8.9–30.3)	82.2 (69.7–91.1)		Referent	Referent
South	45,574 (39,694–51,454)	32.1 (22.8–42.5)	67.9 (57.5–77.2)		−0.83	0.43 (0.19–1.00)
Midwest	33,296 (28,374–38,218)	15.5 (7.3–27.3)	84.5 (72.7–92.7)		0.22	1.25 (0.46–3.39)
West	37,228 (31,038–43,418)	19.6 (9.4–33.8)	80.4 (66.2–90.6)		−0.13	0.88 (0.32–2.40)

*The data source was the National Center for Health Statistics, National Ambulatory Medical Care Survey (*5*). All analyses were conducted by using SUDAAN (https://www.rti.org/impact/sudaan-statistical-software-analyzing-correlated-data) to account for the complex sampling design of the survey. OR, odds ratio; PCMH, patient-centered medical home. †Likelihood determined by using a logistic regression model that included the factors PCMH designation and region of country. β is the coefficient of the variable, indicating direction and strength of the association. ‡PCMH designation of first-listed National Ambulatory Medical Care Survey–sampled office. An estimated weighted total of 149,483 office-based physicians indicated that they evaluate patients for or treat patients with sexually transmitted infections at their first-listed sampled office. PCMH data were missing (i.e., the physician refused to answer, did not know, or left blank or an instrument error occurred) for 10.8% of weighted office-based physicians treating gonorrhea and syphilis. §Estimates might not sum to the estimated weighted total because of rounding of weighted numbers.

Our nationally representative analysis demonstrates that most office-based physicians who provide STI services reported not having on-site access to the recommended injectable medications for gonorrhea and syphilis management. The PCMH designation has been noted as an indicator for quality of care by the American Medical Association (*6*) and thus might be an indication for greater access to more complex treatments and supplies, such as injectable antimicrobial drugs. In addition, previous research has found that PCMHs have larger provider staffs and are affiliated with larger medical groups or health systems that may have access to greater resources, characteristics that might result in these practices having greater access to injectable medications to treat gonorrhea and syphilis patients (*7*). Differences in drug availability by region were not statistically significant; however, we note that the point estimates of on-site availability were highest among physicians in areas with the highest levels of STIs (the southern United States) and where congenital syphilis has been most commonly observed (the western United States) (*1*).

We provide a national estimate of the percentage of US physicians treating patients with STIs that report not having the recommended antimicrobial drugs on-site for same-day treatment of gonorrhea and primary and secondary syphilis. In light of our findings, we believe that future research should focus on determining the factors preventing physicians from providing these medications on-site. The costs of obtaining and carrying these medications, as well as issues of storage and shelf life, should be explored to determine if these factors are barriers. In addition, the implications of prescribing alternative treatments or delaying care in situations when medications are not readily available on-site should be further explored. Mitigating the lack of medication availability to treat these infections will help public health officials stop the rise in STI disease.
